# Monetary Valuation of PM_10_-Related Health Risks in Beijing China: The Necessity for PM_10_ Pollution Indemnity

**DOI:** 10.3390/ijerph120809967

**Published:** 2015-08-21

**Authors:** Hao Yin, Linyu Xu, Yanpeng Cai

**Affiliations:** 1 State Key Laboratory of Environmental Simulation and Pollution Control, School of Environment, Beijing Normal University, No. 19, Xinjiekouwai Street, Haidian District, Beijing 100875, China; 2 Department of Development and Planning, Danish Centre for Environmental Assessment, Aalborg University, Skibbrogade 5, 9000 Aalborg, Denmark; 3 Institute for Energy, Environment and Sustainable Communities, University of Regina, 120, 2 Research Drive, Regina, SK S4S 7H9, Canada. E-Mails: yinhao@mail.bnu.edu.cn (H.Y.); yanpeng.cai@bnu.edu.cn (Y.C.)

**Keywords:** inhalable particulate matter (PM_10_), monetary valuation, health loss, health risk, Beijing

## Abstract

Severe health risks caused by PM_10_ (particulate matter with an aerodynamic diameter ≤10 μm) pollution have induced inevitable economic losses and have rendered pressure on the sustainable development of society as a whole. In China, with the “Polluters Pay Principle”, polluters should pay for the pollution they have caused, but how much they should pay remains an intractable problem for policy makers. This paper integrated an epidemiological exposure-response model with economics methods, including the Amended Human Capital (AHC) approach and the Cost of Illness (COI) method, to value the economic loss of PM_10_-related health risks in 16 districts and also 4 functional zones in Beijing from 2008 to 2012. The results show that from 2008 to 2012 the estimated annual deaths caused by PM_10_ in Beijing are around 56,000, 58,000, 63,000, 61,000 and 59,000, respectively, while the economic losses related to health damage increased from around 23 to 31 billion dollars that PM_10_ polluters should pay for pollution victims between 2008 and 2012. It is illustrated that not only PM_10_ concentration but also many other social economic factors influence PM_10_-related health economic losses, which makes health economic losses show a time lag discrepancy compared with the decline of PM_10_ concentration. In conclusion, health economic loss evaluation is imperative in the pollution indemnity system establishment and should be considered for the urban planning and policy making to control the burgeoning PM_10_ health economic loss.

## 1. Introduction

In recent decades, the economy and population growth in China have come at the expense of environmental degradation [1] and a public health emergency. Chinese life expectancy has decreased by 5.5 years because of the air pollution [2]. PM_10_ (particulate matter with an aerodynamic diameter ≤10 μm), as one of the primary air pollutants, has become a serious issue in recent years as a result of emissions from the fast economic development of transportation, industry and tourism [3,4]. More than three million people are estimated to have died prematurely as a result of outdoor particulate matter pollution in 2010, as reported by the World Health Organization’s Global Burden of Diseases [5]. The great economic losses caused by this pollution cannot be ignored in sustainable national or regional development. PM_10_ health economic loss evaluation could help the decision-makers grasp how much benefit would be associated with PM_10_ pollution control and would also contribute to the establishment of a PM_10_ pollution indemnity system. Here in this study we quantify the associated amount of health-related economic losses to serve as and provide a proxy for the health risks of PM_10_ pollution.

### 1.1. PM_10_-Related Health Impacts and Risks

PM_10_, with an aerodynamic diameter less than 10 μm, can be detrimental for human health because it penetrates deep into the respiratory system and lungs. PM_10–2.5_, the coarse fraction, consists mainly of organic material, silicates and larger carbon aggregates, while the fine fraction is dominated by carbon aggregates and sulfur aerosols [6,7]. Additionally, the coarse fraction provokes higher inflammatory responses than particles in the smaller size ranges, and the fine fraction can penetrate more deeply into the respiratory tree and evoke greater cytotoxicity [8]. Chemical analysis of particles has indicated that heavy metals contained in the particulate matter represent great risks to human body as well [9,10]. Apart from chemical and toxicology analyses, a large body of epidemiological studies over the past decades has indicated that there is a consistent association between exposure to PM_10_ pollution and adverse health effects [11,12,13,14]. Studies showed that the urban areas always suffer more severe PM_10_ pollution compared with suburban or rural areas, could result in highly potential of health risks [15] due to the complexity of the urban environment. For instance, the urban heat island effect influences the particulate matter distribution and composition [16,17]. Numerous studies have focused on the PM_10_-bound PAHs health risk [18,19,20], while it would probably underestimate the health risk potential of PM_10_ mixtures. Epidemiological studies [12,21] could avoid the underestimation by taking the PM_10_ pollution as a whole process during a long-term study.

Under growing scientific and social concerns, the Beijing Environmental Protection Bureau has implemented a series of acts and plans for PM_10_ pollution control, and numerous studies in China have focused on PM_10_ source apportionment [22], spatial distribution [23], cohort studies [24] and meta-analyses [25].

### 1.2. Economic Evaluation of PM_10_-Related Health Impacts

The health impacts resulting from inhalable particles cause a great deal of medical costs and societal losses [26,27], which places significant pressure on environmental managers and decision makers. Economic evaluation of PM_10_-related health risks is a process of monetary valuation, which is the practice of converting measures of non-market goods into monetary units [28]. Additionally, it is an effective tool for the policy making for the environmental pollution indemnity and health risk quantification. Many researchers pursue the balance between fast growing economy and environmental conservation [29,30]; therefore, economic loss evaluation could be an essential and basic research fields for the environmental management and social sustainable development. As a result of the “Polluters Pay Policy” in China, it is essential to focus on the economic losses due to PM_10_-related health effects; the health economic losses can not only illustrate to policy makers/residents in the polluted area how much PM_10_ pollution impacts on health, but can also be employed in sustainable development decision making. In this sense, PM_10_ health economic loss evaluation could be of benefit for the establishment of a PM_10_ pollution indemnity system and imperative for PM_10_ pollution control and management. Thus, the economic losses due to the health risks of inhalable particles command attention.

A study in China monetized the health benefits using value of statistical life (VOSL), which illustrated that air quality improvement prevented about 3442 million Yuan in losses in 2010 in Shanxi Province [31]. By contrast, a city in Malaysia suffered losses of around 1.1–1.7 million dollars annually due to the haze [32] that caused by stagnant meteorological conditions and high particulate matter concentration [33]. Studies elicited health benefits by lowering the ambient particle concentration [34], which would be helpful for the cost-effectiveness of policy making [35] and sustainable urban planning. Based on the GIS system and the monitoring system, researchers evaluated the PM_10_-related health economic losses in China in 2009, which illustrated a general health economic loss across China [21]. Studies showed that the particulate matter health economic losses in China were approximately 1%–5% of the national/regional GDP [36,37]. PM_10_-related health economic losses in 111 cities of China were conducted, covering most of the large and medium-sized cities in China [38], but these studies did not perform detailed research inside the cities. Beijing, as the capital of China, has commanded great attention about the health impacts and losses related to PM_10_ pollution. Most health economic loss studies focus on the short-term effects. Some severe haze events that caused health economic losses were studied in Beijing [39]; whereas long-term studies about the quantification of PM_10_ health effects could be more supportive for policy making over a longer term.

The amended human capital (AHC) approach is based on the human capital approach (HCA), which takes *per capita* GDP as a calculation index rather than the value of statistic life (VSL) [40] to avoid individual differences in productivity loss. The AHC approach has the advantage of conveniently providing data to calculate the health economic losses and is widely accepted by researchers. The cost of illness (COI) approach includes the direct medical costs, the direct nonmedical costs (travel, accommodations, and meal expenses) and indirect costs (medical leave, time off, and productivity loss). Because of data availability, many studies have utilized the AHC/HCA [41] and the COI [27,32] economic methods to estimate the health economic loss due to the diseases. We list the details of the features of each of the methods in Table 1.

**Table 1:** Different economic analysis methods and their characteristics.

Economic Evaluation Method	Advantages	Limitation	Scope of Application
AHC	It can obtain data conveniently; It is simple and clear; it is time-saving and economic for researchers; It reflects human health economic loss in a certain degree.	It cannot reflect the medical costs due to the disease.	It is suited to the chronic diseases that cause absence from work; It is suitable for deaths caused by diseases.
COI	Includes all costs caused by certain diseases, direct medical costs, the direct nonmedical costs and indirect costs reflect the human health loss.	It is limited to short-term health impacts costs estimation.	It is suitable for acute diseases that can be obtained in hospitals.

Studies in recent years have paid more attention on the economic cost caused by particle pollution, while the relationship between economic cost and urban functionality should be studied further. In this study, we aim to combine epidemiological studies with economic methods to estimate the economic cost caused by PM_10_ related health risks in different districts and functional zones in Beijing, China, which could be imperative and meaningful for the urban planning according to the functionality and establishment of a PM_10_ pollution indemnity system.

## 2. Materials and Methodology

In this study, we propose an integrated evaluation method that combines the Amended Human Capital (AHC) approach and Cost of Illness (COI) methods for the health economic loss evaluation in Beijing, China, over a relatively long term scale from 2008 to 2012, focusing on the districts/counties scale, functional-zone scale and city scale. This study provides a scientific basis for urban PM_10_ pollution management and pollution indemnity policy implementation.

### 2.1. Study Area

Beijing, as the capital of China, has a population of more than 20 million, and in 2012, over four million private vehicles were in used Beijing, resulting in serious air pollution problems. Large amounts of air pollutants have been emitted into the atmosphere in Beijing in recent years, especially PM_10_. The PM_10_ concentration exceeds more than 20% of the PM_10_ second-grade ambient air quality standard of China (annual concentration is 70 μg/m^3^ according to the *China Ambient Air Quality Standard GB 3095-2012*) [42] in recent years.

**Figure 1:**
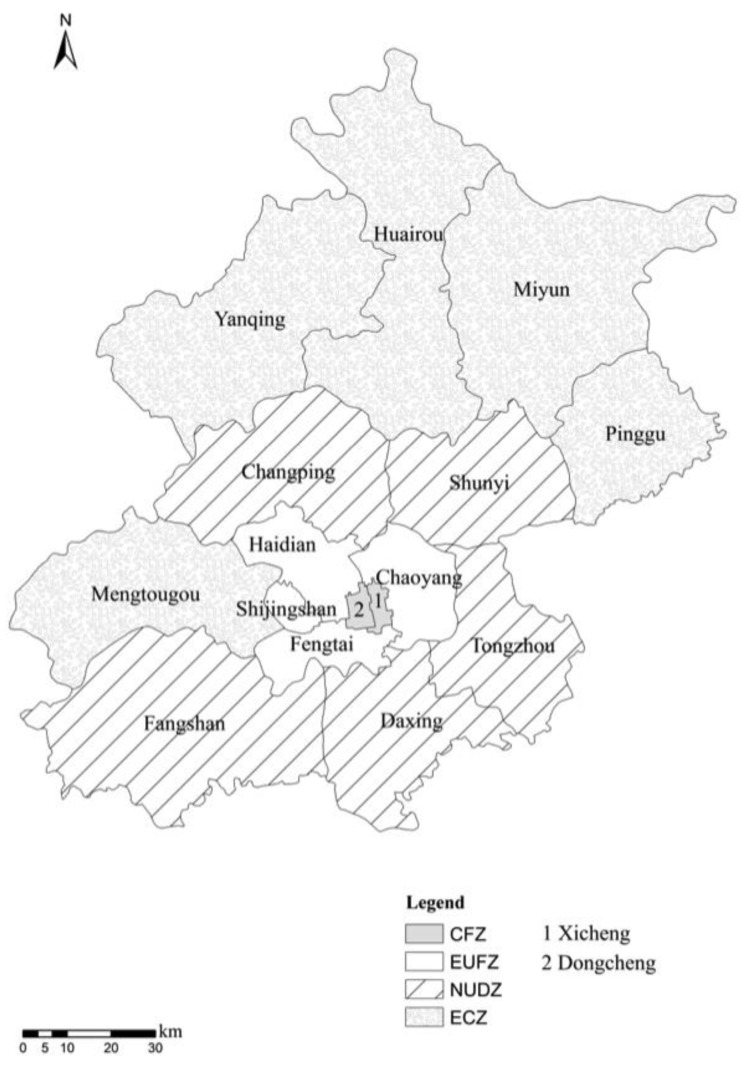
Functional regionalization of Beijing.

To optimize urban layout and planning, the municipality of Beijing conducted a functional regionalization in 2006, classifying 16 districts and counties of Beijing into four functional regions (Figure 1) listed as follows:

(1) The “core functional zone” (CFZ) includes the Dongcheng and Xicheng districts. The main task of this region is to strengthen city management, protect the ancient style of Beijing, improve the living environment, and develop the modern service industry.
(2) The “expanding urban function zone” (EUFZ) consists of the Chaoyang, Haidian, Fengtai and Shijingshan districts. The function of this region is to expand the nationwide and worldwide export-oriented economical service function, promote scientific and technological innovation and develop high-tech industries.
(3) The “new urban development zone” (NUDZ) includes the five districts of Tongzhou, Daxing, Shunyi, Fangshan and Changping. The region relies on the development of the Beijing manufacturing industry and modern agriculture. Additionally, it is also an important area to relocate industry and population from the city center and is the future economic center of Beijing.
(4) The “ecological conservation zone” (ECZ) includes the five counties of Mentougou, Pinggu, Huairou, Miyun and Yanqing. This region is essential for guaranteeing the regional sustainable development of Beijing.

With diverse environmental and economic conditions, different functional zones have various regional development goals and urban functions, which influences the regional air quality and residents accordingly.

### 2.2. PM_10_-Related Health Risk and Impact Quantification Methods

#### 2.2.1. Hazard Identification

In this study, we selected the main health endpoints that have been widely used in many previous studies [25,38,43], including individual mortality, chronic bronchitis, respiratory hospitalization, cardiovascular hospitalization, outpatient visits to internal medicine, outpatient visits to pediatrics, acute bronchitis and asthma attacks.

#### 2.2.2. Health Risk and Impact Assessment Method

The incidence of all the health endpoints caused by PM_10_ is a low-probability event in a given population, which conforms to Poisson’s law. The previous studies indicated that there was an approximately linear exposure-response relationship between PM_10_ and related health impacts [44,45,46]. Based on this, this study utilized a linear exposure-response model (Equation (1)), which is widely used in many studies related to the costs and benefits evaluation [38,43,47]. The exposure-response function of particulate matter is based on epidemiological studies linking inhalable particulate pollution with relative health endpoints in the form of a Poisson regression (Equation (2)):

(1) $${\text{E}}_{\text{i}}={\text{E}}_{0\text{i}}\times \left[1+{\text{β}}_{\text{i}}\times \left(\text{C}-{\text{C}}_{0}\right)\right]$$

(2) $${\text{N}}_{\text{i}}=\text{P}\times {\text{E}}_{\text{i}}\times \left[1-\frac{1}{\text{EXP}\left({\text{β}}_{\text{i}}\times \left(\text{C}-{\text{C}}_{0}\right)\right)}\right]$$

where E represents the relative risks of different health endpoints caused by increase of 1 mg/m^3^ PM_10_, E_0_ is the baseline incidence of certain health endpoints, β means the exposure-response coefficient of certain health endpoint, which refers to the change in the concentration of PM_10_ per 1 mg/m^3^, the corresponding change in the proportion of certain health risk, C (mg/m^3^) is the concentration of different districts or counties in Beijing, C_0_ (mg/m^3^) is the threshold concentration with no observed health impact of PM_10_ below this level, taking the average year guiding value set by WHO, *i.e.*, PM_10_ is 0.02 mg/m^3^ in this study, P represents the exposure population in the study area, and ${\text{N}}_{\text{i}}$ means the population with health endpoint. Some health endpoints such as changes in lung function and restricted days caused by PM_10_ pollution are excluded from the calculation because of the unavailability of data.

#### 2.2.3. Exposure-Response Coefficients Selection

To estimate the PM_10_ health impacts, the exposure-response coefficients β, and baseline frequencies (E_0_) were mainly collected from domestic studies to make the evaluation results more authentic (Table 2).

**Table 2:** PM_10_ related health impacts exposure-response coefficients β.

Health Endpoints	β (95% CI)	E_0_
Individual mortality	4.3 (2.60, 6.10) [13,14]	0.01013 [48]
Chronic bronchitis	5.77 (1.93, 9.61) [49]	0.01390 [50]
Respiratory hospitalization	1.2 (0.80, 1.60) [51]	0.01022 [52]
Cardiovascular hospitalization	0.7 (0.30, 1.10) [51]	0.00546 [52]
Outpatient visits to internal medicine	0.01374 (0.01077, 0.01679) [53]	0.41105 [52]
Outpatient visits to pediatrics	0.01551 (0.01041, 0.02060) [53]	0.15300 [52]
Acute bronchitis	5.5 (1.89, 9.11) [49]	0.03800 [52]
Asthma attacks	3.9 (1.90, 5.90) [54]	0.05610 [55]

### 2.3. Monetary Valuation Model of PM_10_-Related Health Risks

In this study, we utilized the Amended Human Capital (AHC) approach for mortality and chronic disease cost calculation and the Cost of Illness (COI) approach for other morbidity costs in the short term based on the health risks and impacts calculated in Section 2.3. The amended human capital approach is revised based on the human capital approach that encompasses a societal perspective and estimates an individual’s contribution to society by applying labor force earnings as a measure of productivity. Nevertheless, the traditional human capital approach assumes that different people embody different life values; this approach causes great controversy as an ethical issue. As a result of this, the AHC approach has tended to be widely used in recent decades because it uses per capita GDP to measure the value of a statistical year of life. It can be viewed as a social statement of the value of avoiding premature mortality and estimates human capital (HC) from the perspective of the entire society, neglecting individual differences [21]. For individual mortality, we calculated the annual human capital multiplied by the years of premature mortality and population as shown by the following function (Equations (3)–(4)). In terms of chronic bronchitis, we consider that people would lose 32% of their working ability [39], which means they could not work as effective as if they were healthy (Equation (5)):

(3) $$\text{HCL}=\overset{t}{\underset{y=1}{\sum }}GDP_{Py}^{dv}=GDP_{Pc0}\cdot \overset{t}{\underset{y=1}{\sum }}\frac{{\left(1+\text{α}\right)}^{y}}{{\left(1+r\right)}^{y}}$$

(4) $${\text{TL}}_{\text{m}}=HCL_{m}\times {\text{P}}_{1}$$

(5) $${\text{TL}}_{\text{b}}=\text{δ}\times HCL_{m}\times {\text{P}}_{2}$$

where HCL is the amended human capital loss *per capita* caused by PM_10_; *t* is the average number of life-years lost as a result of PM_10_ pollution, average 18 years; $GDP_{Pci}^{dv}$ is the *per capita* GDP discounted value in the number y*th* year in Beijing; $GDP_{Pc0}$ is the *per capita* GDP in a basic year in Beijing; α is the *per capita* GDP growth rate; *r* is the social discount rate; TL_m_ is the economic total loss of excess mortality; TL_b_ is the economic loss of chronic bronchitis; P_1_ is the number of excess deaths; P_2_ is the number of chronic bronchitis patients and δ is the disability weights resulting from chronic bronchitis disease (32%). Here, the GDP growth rate and social discount rate are 10% and 8%, respectively, according to the recent years’ economic development.

The COI approach calculates the medical and economic burden that a disease may have on the society as a whole. Due to the consideration that the medical expenses of chronic diseases are difficult to collect, the COI approach is mainly used for counting the costs of short-term diseases. We calculated the per case cost of hospitalization, outpatient visits to internal medicine, outpatient visits to pediatrics, acute bronchitis and asthma attacks (Table 3). The economic loss because of the disease medical costs would be calculated according to Equation (6):

(6) $$Disease\;Loss\;\left(DL\right)=\left(\;MC_{i}+HCL\times T_{i}\right)\times P_{i}$$

where *MC_i_* represents the medical costs per case in a certain disease (i) and *T_i_* means the time of absence from work as a result of a certain disease (i). Here, respiratory diseases result in approximately 10.6 days of hospitalization or rest, and asthma would take approximately 6.9 days; these times were obtained from statistical yearbooks [56]. *P_i_* is the population that has a certain disease (i).

**Table 3:** Estimated medical costs per case of different health endpoints.

Year	Medical Costs ($)			
	Hospitalization	Asthma Attacks	Acute Bronchitis	Outpatient Visits
2008	2233.94	255.54	406.77	52.41
2009	2595.36	293.67	434.19	54.88
2010	2650.59	319.90	463.47	57.35
2011	2705.81	346.13	494.72	59.82
2012	2761.04	372.36	528.08	59.82

### 2.4. Data Collection

**Figure 2:**
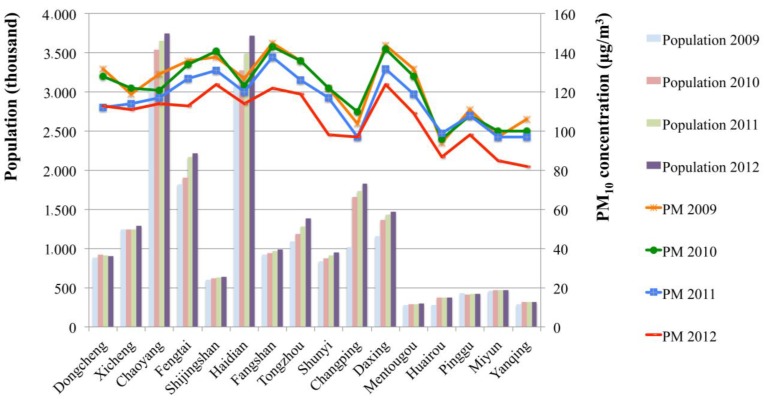
Annual PM_10_ concentration and population of different districts and counties in Beijing from 2009 to 2012.

Annual general PM_10_ pollution concentrations of Beijing from 2008 to 2012 and PM_10_ concentrations of 16 districts and counties from 2009 to 2012 were obtained from the Beijing Environmental Statement (2008–2012) [57]; PM_10_ concentrations in different districts/counties were missing for 2008. The annual PM_10_ concentrations in Beijing from 2008 to 2012, which were 122, 121, 121, 114 and 109 μg/m^3^, respectively, were higher than second-grade ambient air quality standard of China (annual concentration is 70 μg/m^3^ according to the *China Ambient Air Quality Standard GB 3095-201*) [42]. Beijing GDP per capita was $10,493, $10,891, $12,016, $13,286, $14,170 dollars, respectively, from 2008 to 2012; these data were collected from the Beijing statistical yearbooks from 2008 to 2012. For different districts and counties, the PM_10_ concentration and population data, which were obtained from the Information Statistics Bureau websites, are illustrated in Figure 2.

## 3. Results and Discussion

### 3.1. PM_10_-Related Health Risks in Beijing

Using Equation (1), we calculated relative risks of various health endpoints, and the results showed that individual mortality, chronic bronchitis, respiratory hospitalization, acute bronchitis and asthma attacks slowly decreased from 2008 to 2012 in Beijing, while cardiovascular hospitalization, outpatient visits to internal medicine and outpatient visits to pediatrics showed a generally stable trend (Table 4). Outpatient visits to internal medicine, outpatient visits to pediatrics and asthma attacks showed relatively higher risks among all the health endpoints related to PM_10_ pollution.

### 3.2. PM_10_-Related Health Impacts of Different Districts in Beijing

PM_10_-related health impacts from 2008 to 2012 were calculated according to Equation (2) based on the results of PM_10_-related health risks. The health impacts from 2008 to 2012 are shown in Table 5. The total health impacts rose slowly from 2008 to 2010 and then showed a decreasing tendency until 2012. In 2010, the total health impacts were the highest in the study period. The health impacts started to decrease in 2011, whereas the PM_10_ annual concentration decreased from 2010 to 2012. Asthma attacks and acute bronchitis accounted for the relatively larger portion of the total health impacts compared with other health endpoints; however, it should not be ignored that individual mortality is still quite large among the health impacts.

**Figure 3:**
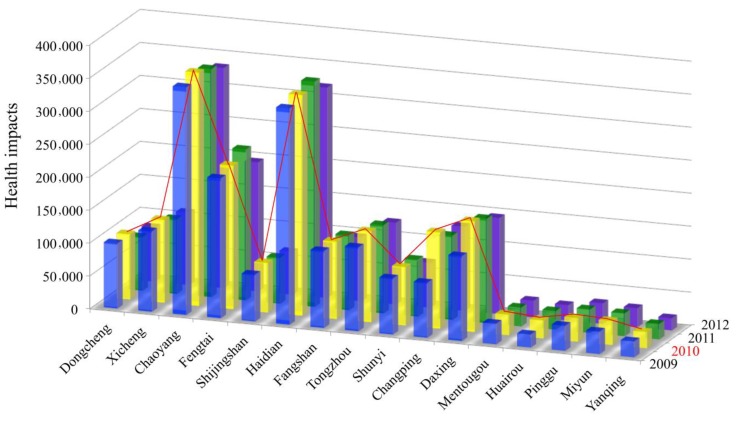
Health impacts of different districts and counties.

The health impacts of different districts and counties were analyzed from 2009 to 2012 (Figure 3), which showed that Chaoyang, Haidian and Fengtai had in the most serious health impact situations compared with other districts and counties (Figure 3). Larger population density and relatively high PM_10_ concentration made great contribution to the serious health impacts in these districts. It was clear that the Mentougou, Huairou, Pinggu, Miyun and Yanqing districts showed the lowest health impacts among all the districts. These districts and counties with relative good environmental quality and natural resources, relatively smaller population, thus the health impacts is lower than other districts.

**Table 4:** PM_10_-related health risks from 2008 to 2012.

Health Endpoints	Frequencies (95% CI)				
	2008	2009	2010	2011	2012
Individual mortality	0.0093 (0.0082–0.0105)	0.0093 (0.0081–0.0104)	0.0093 (0.0081–0.0104)	0.0091 (0.0080–0.0101)	0.0089 (0.0079–0.0100)
Chronic bronchitis	0.0203 (0.0157–0.0249)	0.0202 (0.0157–0.0248)	0.0202 (0.0157–0.0248)	0.0198 (0.0156–0.0240)	0.0195 (0.0155–0.0235)
Respiratory hospitalization	0.0115 (0.0111–0.0119)	0.0115 (0.0110–0.0119)	0.0115 (0.0110–0.0119)	0.0114 (0.0110–0.0118)	0.0113 (0.0109–0.0117)
Cardiovascular hospitalization	0.0059 (0.0056–0.0061)	0.0058 (0.0056–0.0061)	0.0058 (0.0056–0.0061)	0.0058 (0.0056–0.0060)	0.0058 (0.0056–0.0060)
Outpatient visits to internal medicine	0.4116 (0.4115–0.4118)	0.4116 (0.4115–0.4117)	0.4116 (0.4115–0.4116)	0.4116 (0.4115–0.4117)	0.4116 (0.4114–0.4117)
Outpatient visits to pediatrics	0.1532 (0.1532–0.1533)	0.1532 (0.1532–0.1533)	0.1532 (0.1532–0.1533)	0.1532 (0.1531–0.1533)	0.1532 (0.1531–0.1533)
Acute bronchitis	0.0593 (0.0453–0.0733)	0.0591 (0.0453–0.0730)	0.0591 (0.0453–0.0730)	0.0576 (0.0448–0.0705)	0.0566 (0.0444–0.0688)
Asthma attacks	0.0784 (0.0670–0.0899)	0.0782 (0.0669–0.0895)	0.0782 (0.0669–0.0895)	0.0767 (0.0661–0.0872)	0.0756 (0.0656–0.0856)

**Table 5:** PM_10_-related health impacts from 2008 to 2012.

Health Endpoints	Number of Cases				
	2008	2009	2010	2011	2012
Individual mortality	55,844	57,196	63,910	60,800	58,675
Chronic bronchitis	126,528	129,592	144,803	137,745	132,921
Respiratory hospitalization	22,400	22,954	25,648	24,493	23,706
Cardiovascular hospitalization	6833	7003	7825	7482	7248
Outpatient visits to internal medicine	9771	10,018	11,194	10,726	10,406
Outpatient visits to pediatrics	4106	4210	4704	4507	4373
Acute bronchitis	431,698	442,139	494,037	469,848	453,285
Asthma attacks	436,235	446,815	499,261	475,068	458,547
All	1,093,415	1,119,927	1,251,383	1,190,669	1,149,161

**Figure 4:**
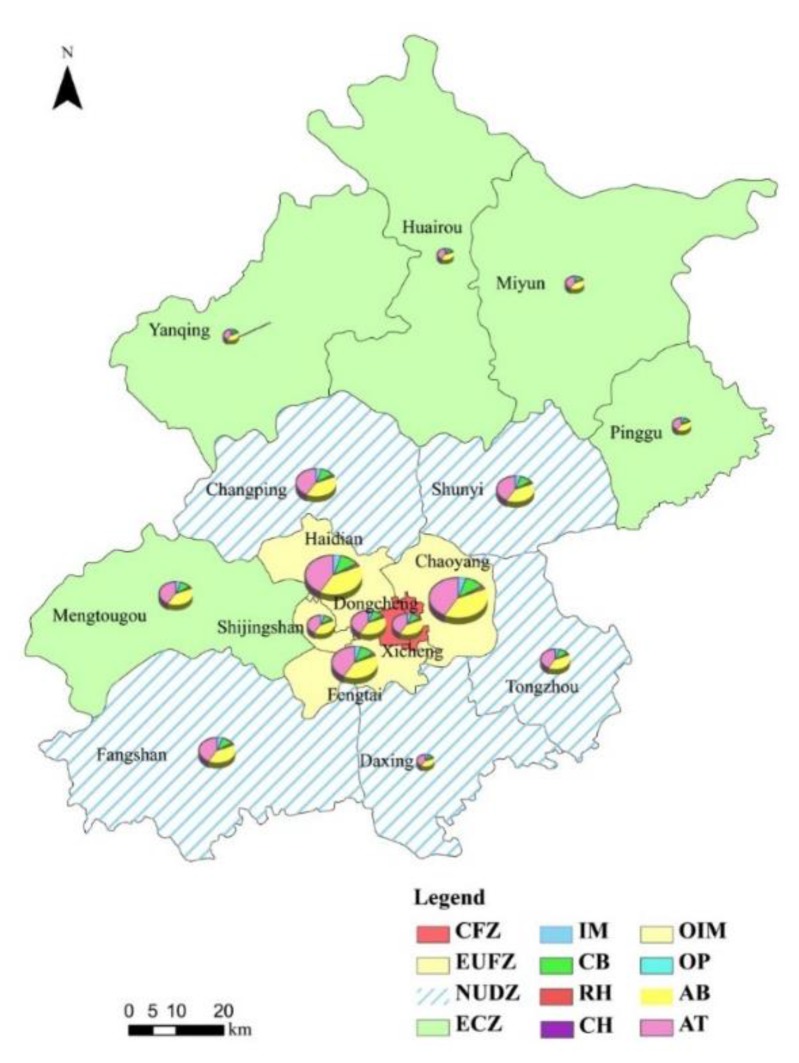
Different health impacts in different districts and counties in 2010. Note: In Figure 4, IM, CB, RH, CH, OIM, OP, AB and asthma attacks refer to individual mortality, chronic bronchitis, respiratory hospitalization, cardiovascular hospitalization, outpatient visits to internal medicine, outpatient visits to pediatrics, acute bronchitis, asthma attacks, respectively. The size of the pie chart refers to the sum of various health impacts.

We also calculated different health impacts in all the districts during the study period, which illustrated that asthma attacks, acute bronchitis and chronic bronchitis cases represented the largest proportion of the total health impacts in most districts of Beijing from 2009 to 2012. Because the health impacts displayed similar proportions every year during the study period, we took the results from 2010 that showed the largest health impacts during the study period, as an example that is shown in Figure 4.

Overall, health impacts were much higher in the Chaoyang, Haidian and Fengtai districts from 2009 to 2012. In terms of health endpoints, asthma attacks and acute bronchitis made up a large part of the total health impacts in the study period.

### 3.3. Monetary Costs of PM_10_-Related Health Impacts in Beijing

The economic loss of various health endpoints was calculated based on the above analysis and is shown in Table 6. In 2008, the health economic loss was approximately US $ 23.8 billion, which was the lowest loss among the five years. During the study period, the economic losses increased relatively quickly from around 23.6 to 31.2 billion dollars, while the rising trend slowed down from 2010 to 2012. In terms of the various health endpoints, the economic losses increased with a similar trend as the total loss. Among all of the health endpoints, individual mortality and chronic bronchitis made up the largest portion of the economic losses and represented approximately 98% of the total losses.

**Table 6:** Economic loss of PM_10_-related health impacts from 2008 to 2012.

Health Endpoints	Economic Loss ($)				
	2008	2009	2010	2011	2012
Individual mortality	1.35E + 10	1.41E + 10	1.71E + 10	1.77E + 10	1.79E + 10
Chronic bronchitis	9.82E + 09	1.03E + 10	1.24E + 10	1.28E + 10	1.30E + 10
Respiratory hospitalization	6.12E + 07	7.06E + 07	7.96E + 07	7.70E + 07	7.49E + 07
Cardiovascular hospitalization	1.87E + 07	2.15E + 07	2.43E + 07	2.35E + 07	2.29E + 07
Outpatient visits to internal medicine	5.15E + 05	5.54E + 05	6.37E + 05	6.26E + 05	6.23E + 05
Outpatient visits to pediatrics	2.17E + 05	2.33E + 05	2.68E + 05	2.63E + 05	2.62E + 05
Acute bronchitis	1.04E + 07	1.15E + 07	1.39E + 07	1.40E + 07	1.42E + 07
Asthma attacks	1.20E + 08	1.38E + 08	1.66E + 08	1.67E + 08	1.71E + 08
Total loss	2.36E + 10	2.46E + 10	2.98E + 10	3.08E + 10	3.12E + 10

### 3.4. Discussion

#### 3.4.1. Monetary Valuation Based on Different Districts in Beijing

**Figure 5:**
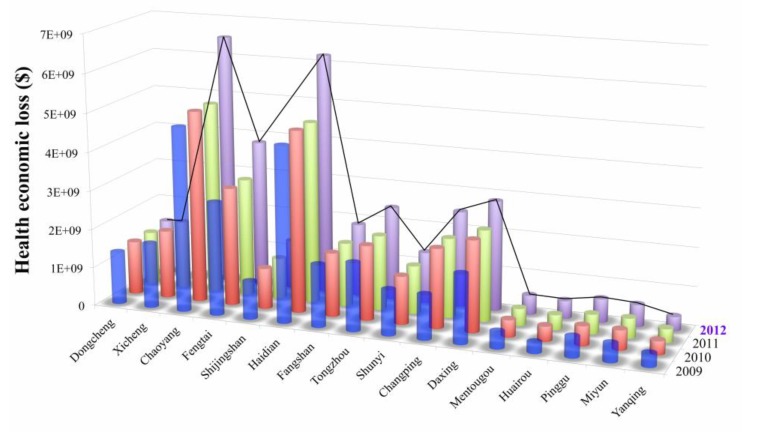
Health economic loss in different districts and counties in 2012.

From 2009 to 2012, the economic losses due to PM_10_-related health impacts maintained a slowly increasing trend that was especially rapid in the Chaoyang, Haidian, Fengtai, Tongzhou, Fangshan, Shunyi, Changping and Daxing districts (Figure 5). Nevertheless, economic losses in Mentougou, Huairou, Pinggu, Miyun and Yanqing was relatively smaller compared with other districts.

Among all the health endpoints, individual mortality and chronic bronchitis accounted for the largest portion of the total economic losses, which was about 98% of the total loss during the study period.

**Figure 6:**
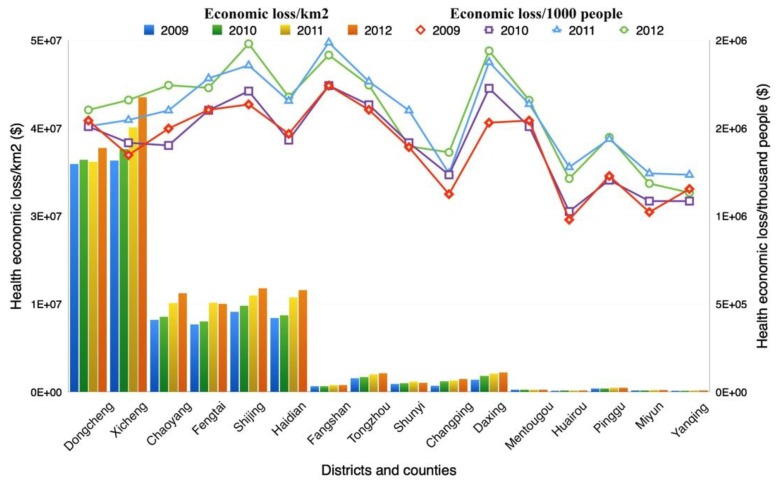
Health economic loss of per km^2^ and per thousand people in different districts.

Economic loss per km^2^ was quite different from the total loss of the districts (Figure 6). Among all of the districts/counties, Xicheng and Dongcheng suffered the highest health economic loss/km^2^, ranging from 32.40 to 41.88 million dollars. Additionally, the Chaoyang, Haidian, Fengtai and Shijingshan districts had similar economic losses of 9.56 to 14.71 million dollars/km^2^. Huairou, Yanqing, Mentougou and Pinggu had the smallest health economic loss/km^2^. Overall, in most districts, the health economic loss/km^2^ showed a slowly rising trend from 2009 to 2012.

In terms of the health economic loss per thousand people (Figure 6), it fluctuated in different districts and counties and inter-annual variances were quite significant during the study period. It ranged from around US $ 9.8E + 05 to US $ 2.0E + 06 in different districts from 2009 to 2012. Overall, Huairou showed the lowest economic loss, while it was much higher in Fangshan, Shijingshan and Daxing compared with the other districts. Meanwhile, the health economic loss/thousand people increased slightly in most districts since 2009 in general, except the Shunyi, Yanqing districts, *etc*.

Considering the circumstances above, the Chaoyang, Haidian, Fengtai, Dongcheng, Xicheng, Shijingshan and Fangshan districts should take more targeted measures to control the regions’ PM_10_ pollution for their high economic loss in total, per km^2^ or *per capita*. This is of great importance for controlling the total health economic loss as well as the loss per km^2^/*per capita*.

#### 3.4.2. Monetary Valuation Based on Different Functional Zones in Beijing

We analyzed the health economic loss of different functional regions based on the results of the health economic losses of different districts and counties. The results showed that the expanding urban function zone suffered the largest economic losses, ranging from 13.1 to 17.7 billion dollars approximately, which was about 51% of the total economic losses. The new urban development zone’s losses were less (around 29%), ranging from approximately 7.4 to 10.9 billion dollars from 2009 to 2012. There are only two districts in the core functional zone representing 0.5% of the total area; nevertheless, the economic losses of this zone were approximately 3.0 to 3.6 billion dollars, which is around 12% of the total economic losses. In the CFZ and the EUFZ, there was a dramatic increase in the PM_10_ health economic losses from 2009 to 2012. On the contrary, the health economic losses decreased in the NUDZ and ECZ in 2012.

Different functional zones have various development goals, which promote the functional zones’ revealed diverse social, economic and environmental conditions. Urban vegetation improves air quality by influencing pollutant deposition because most plants have a large surface area per unit volume, increasing the probability of deposition compared with the smooth, manufactured surfaces present in urban areas [58]. Studies showed that the CFZ and EUFZ are covered by impervious surfaces and have very little green space [59], which means the emitted airborne particles could not deposit effectively. Moreover, in the CFZ and EUFZ, the population density is 23,602 and 11,362 people/km^2^, which is much higher than the population density in NUEZ (1161 people/km^2^) and ECZ (237 people/km^2^). In this sense, CFZ and EUFZ showed relatively higher PM_10_ pollution intensity (population multiplied by PM_10_ concentration). Compared with this situation in CFZ and EUFZ, the NUDZ and ECZ showed relatively lower pollution intensity and higher amounts of green space, consequently, lower health impacts and economic losses occurred in these functional zones. As a result of this, the PM_10_ health economic losses could be associated with the regional development tendency and environmental conditions and one could take effective measures to moderate the economic losses related to PM_10_ pollution accordingly.

Although the core functional zone showed a lower health economic loss than the EUFZ and NUDZ, the health economic loss per km^2^ was the largest among all of the functional zones, ranging from 32.68 to 38.62 million dollars approximately, which was twice the loss in the expanding urban functional zone. The health economic loss was relatively low per km^2^ in the new urban development zone and ranged from 1.31 to 1.92 million dollars from 2009 to 2012. The health economic loss in the ecological conservation zone was the lowest compared with other functional zones, at approximately 0.3 million dollars per km^2^.

#### 3.4.3. Overall Monetary Valuation in Beijing

**Figure 7:**
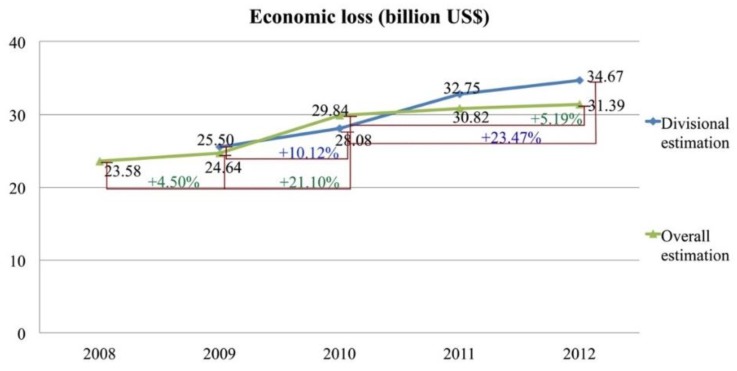
PM_10_ health economic losses of Beijing from 2008 to 2012.

We compared the results obtained at the district and city scales (Figure 7). The health economic losses caused by airborne particle pollution were serious in these years, and the economic losses were approximately 20 to 30 billion dollars every year in Beijing, which accounted for 10%–13% of Beijing’s regional GDP. In the comparison of divisional and overall estimation, we can see that the health economic losses showed different increasing tendencies (Figure 7), which illustrated that the economic losses would be influenced by the spatial differences and social/economic factors, not only the particle concentrations but also population density, GDP *per capita*, medical costs, *etc*.

The divisional estimation showed a relatively faster increasing trend (Figure 7), which is probably due to the fact that economic development and population density in the central area of the city grew more quickly than in the suburban districts. Among the four functional zones, only the ECZ sets environmental and ecological goals for its future development, while the other three zones focus on the economic development, which results in great economic losses in those regions. Therefore, urban functional zones should consider environmental protection and conservation objectives for their sustainable development in the long run.

#### 3.4.4. PM_10_ Pollution Control Policies in Beijing from 2008 to 2012

From 2008 to 2012, the PM_10_ concentration dropped to a certain extent from 122 to 109 μg/m^3^ as a result of the air pollution control policies implemented in Beijing. From 2008 to 2012, Beijing took various countermeasures to combat the air pollution to make some progress in addressing the issue.

The municipality imposed strict regulations, mainly concerning road dust, vehicles, industrial emissions and coal combustion. These regulations were enforced in Beijing and also in the cities around it. In 2011, specific goals were set for PM_10_ pollution control instead of general air pollution regulation. To make more progress, the Beijing municipality started monitoring PM_10_ and PM_2.5_ concentrations in an intensive network; this monitoring was supportive for particulate matter pollution control and management in 2012. The government weeded out 400 thousand old vehicles that could not meet the environmental standards and also decreased coal consumption by 62%. Overall, the policies to combat PM_10_ pollution in Beijing are becoming more stringent in recent years and are resulting in great contributions to PM_10_ pollution control. Nonetheless, with the serious PM_10_ pollution situation in Beijing, more applicable measures should be taken to combat the issue.

According to the former studies, pollutant emission control from thermal power plants according to the national policy could be helpful for ambient particle pollution reduction [60] and more use of anthracite could reduce the emission of air pollutants [61]. Additionally, the government needs to control vehicle usage and encourage clean energy consumption and less coal combustion, *etc*. The functional zone evaluation results showed that it is essential for the government and environmental managers to set specific environmental goals for functional planning. Based on the PM_10_ health economic loss evaluation results, economic loss evaluations could be meaningful for the pollution control and pollution indemnity.

## 4. Conclusions

In this study, we assessed inhalable particulate matter pollution including health risks, impacts and economic losses in Beijing from 2008 to 2012 with the AHC and COI approaches on the district scale, functional-zone scale and city scale based on the epidemiological studies.

Monetary valuation of PM_10_-related health risks in Beijing utilized authentic data and revised some deficiencies in previous studies for the calculation and analysis, whereas there were still some uncertainties we could not avoid thoroughly. We tried to focus on the main health endpoints and divide the health endpoints into three categories to avoid double counting economic losses. It is impossible to tally all of the relative health endpoints in the study to obtain the total economic losses because of complicated health outcomes due to the PM_10_ pollution; therefore, the calculation of PM_10_ health economic losses in this study is relatively conservative and probably lower than the real losses. Additionally, in this study, we utilized annual PM_10_ concentration data of different functional zones to analyze the correlations between pollution, health risks, economic loss and urban functionality in a relative long-term exposure assessment background. Notwithstanding the generalized data, it is relatively reasonable to obtain real results about the variation of PM_10_ pollution and risk related economic losses in different functional zones. Although there would be a discrepancy in the evaluation, the PM_10_ health economic losses were tremendous, not only for individuals, but also for the whole society and should not be ignored in urban/region sustainable development planning.

From 2008 to 2012, the PM_10_ concentration dwindled slightly; however, the health impacts did not decrease until 2011. Moreover, the health economic losses increased slightly from 2008 to 2012. It was indicated that there was a time lag between the pollution, health impacts and the decreasing health economic losses tendency. This is mainly because that the health economic losses are calculated based on the human capital losses and cost of illness. When the GDP *per capita* and medical costs increase, thereafter, the human capital losses and cost of illness would increase accordingly. Similarly, if the population increases, the PM_10_ exposure population increases as well, which leads to the growth of number of people with a certain health endpoint.

The valuation results could be meaningful for PM_10_ pollution indemnity, because polluters should carry the penalty price of billions of dollars according to the “Polluters Pay Policy”. Regions’ functionality greatly affects the health impacts and economic losses, and in this sense, the regional development should not ignore the environmental goals to control the PM_10_-related health economic losses in China. Moreover, the economic loss control with the current economic development conditions and control policies in Beijing was not effective enough. Therefore, we propose that the PM_10_ health economic loss evaluation results, as the fundamental study, should be considered during the PM_10_ pollution control and pollution indemnity policy-making process.
